# Pediatric multi-drug-resistant tuberculosis in Germany – diagnostic and therapeutic challenges of an “orphan disease”

**DOI:** 10.1007/s00431-023-05167-x

**Published:** 2023-09-14

**Authors:** Hannah-Lena Schäfer, Michael Barker, Peter Follmann, Annette Günther, André Hörning, Petra Kaiser-Labusch, Sebastian Kerzel, Christoph Maier, Samra Roth, Christian Schmidt, Katharina Schütz, Florian Stehling, Marie Struffert, Nina Timmesfeld, Paul Vöhringer, Folke Brinkmann

**Affiliations:** 1 Department of Pediatric Pulmonology, Ruhr University Bochum, St. Josef Hospital, University Hospital of Pediatrics and Adolescent Medicine, Alexandrinenstraße 5, Bochum, 44791 Germany; 2 Department of Pediatrics, Heckeshorn Lung Unit, Helios Klinikum Emil von Behring, Berlin, Germany; 3 Klinik für Kinder- und Jugendmedizin, Westpfalz-Klinikum, Kaiserslautern, Germany; 4 University Hospital Erlangen, Pediatrics, Germany; 5 Professor-Hess-Children’s Hospital, Bremen-Mitte, Germany; 6 Department of Pediatric Pneumology and Allergy, University Children’s Hospital Regensburg, Campus St. Hedwig, Regensburg, Germany; 7 Klinik für Kinder- und Jugendmedizin, St. Vinzenz-Hospital, Dinslaken, Germany; 8 Klinik für Pädiatrische Pneumologie, Allergologie und Neonatologie, Medizinische Hochschule Hannover, Hannover, Germany; 9 Centre for Pediatrics, University Hospital Essen, Essen, Germany; 10 Department of Medical Informatics, Biometry and Epidemiology, Ruhr University, Bochum, Germany; 11 Franz-Lust-Klinik für Kinder- und Jugendmedizin Städtisches Klinikum, Karlsruhe, Germany; 12 Division of Pediatric Pulmonology and Allergology, German Center for Lung Research (ARCN, DZL), University Children’s Hospital, Luebeck, Germany

**Keywords:** Multidrug resistance (MDR), Tuberculosis, Children, Multicenter, Germany

## Abstract

Delay in diagnosing multidrug-resistant tuberculosis (MDR-pTB) in children prolongs time to effective treatment. Data on risk factors for pediatric MDR from low-incidence countries are scarce. Retrospective nationwide case–control study to analyze MDR-pTB cases in Germany between 2010 and 2020 in comparison to a drug-susceptible (DS)-pTB group. We included 52 MDR cases (24 tuberculosis (TB), 28 TB infection (TBI); mean age 7.3 years) and 56 DS cases (31 TB, 26 TBI; mean age 7.9 years). Groups were similar for sex, household size, and migration background. Compared to the DS group, more children with MDR were born in the Commonwealth of Independent States (CIS) (22% MDR-pTB vs. 13% DS-pTB, n.s.) and had more MDR index cases (94% MDR-pTB, 5% DS-pTB, *p* < 0.001). The interval between first healthcare contact and initiation of effective therapy was significantly longer in MDR-pTB (47 days) than in DS-pTB (11 days, *p* < 0.001), correlating with disease progression. Treatment for MDR-pTB was successful in 74%, but 22% experienced long-term adverse effects (e.g., hepatopathy, hearing loss).

*Conclusions*: Close contact to MDR cases or birth in MDR-TB-high-incidence countries are risk factors for MDR-pTB. Early identification of potential MDR index cases by contact investigation, and susceptibility testing in children from high-burden MDR-TB countries are essential for timely diagnosis and treatment, reducing the severity of disease and treatment side effects.

*Trial Registration*: Deutsches Register Klinischer Studien (https://www.drks.de/drks_web/navigate.do?navigationId=trial.HTML&TRIAL_ID=DRKS00023817), DRKS00023817, 2020–09-08.

**Table:** 

**What is Known:** *•Management of children with MDR-TB remains challenging due to difficulties in diagnosing MDR-TB (lack of information on MDR index case, lack of microbiological confirmation in paucibacillary disease).* *•Choice of treatment regimen and monitoring of side effects.*	
**What is New:** *•Children with an MDR-TB index or born in a MDR-TB-high-incidence country are at higher risk of developing MDR-TB in a low incidence country.* *•The time lag to initiate treatment in MDR-TB is longer than in DS-TB and MDR-TB treatment involves a higher risk of adverse effects in longer treatment regimens especially with injectables.*

**What is Known:**

*•Management of children with MDR-TB remains challenging due to difficulties in diagnosing MDR-TB (lack of information on MDR index case, lack of microbiological confirmation in paucibacillary disease).*

*•Choice of treatment regimen and monitoring of side effects.*

**What is New:**

*•Children with an MDR-TB index or born in a MDR-TB-high-incidence country are at higher risk of developing MDR-TB in a low incidence country.*

*•The time lag to initiate treatment in MDR-TB is longer than in DS-TB and MDR-TB treatment involves a higher risk of adverse effects in longer treatment regimens especially with injectables.*

## Introduction

According to the World Health Organization (WHO) [1] and the German Association of the Scientific Medical Societies (AWMF) [2] guidelines, active tuberculosis disease (TB) is defined as clinical, microbiological, or radiological pathology suggestive of tuberculosis [1–5].

Germany is a low-incidence country with 4,127 TB cases in 2020 (5 per 100,000), of which 163 were under the age of 15 years. Pediatric TB incidence in 2020 (1.4 per 100,000) was lower than in previous years, highest under the age of five (2.1 per 100,000) [6].

The WHO defines multidrug-resistant TB (MDR-TB) as resistant to rifampin and isoniazid [1]. In 2019, 2% of all children with pediatric TB (pTB) in Germany had MDR-pTB [7]. Adults with drug-susceptible TB (DS-TB) can develop into MDR-TB by secondary drug resistance, whereas children with infection by resistant strains almost exclusively acquire these from adult index cases [5, 8]. Due to frequently missing reference to index patients and the absence of sensitive diagnostic tools, time-to-treatment for MDR-pTB is often extended [9, 10]. Repeated microbiologic sampling is essential for diagnosing children, particularly in those with paucibacillary [8, 11] or extrapulmonary disease [12]. Drug susceptibility testing (DST) is commonly phenotypic and depends on microbiological culture. Genotypic detection of mutations is not routinely used [9, 13]. The challenging diagnostic process and lack of contact investigations delays administering appropriate treatment [10, 14].

Children with MDR-pTB are treated with anti-TB drugs adapted to their index resistance pattern [1, 4, 8, 11, 15]. Pharmacokinetic and pharmacodynamic data in children are limited, and recommended regimens changed twice during the study period. Recent WHO guidelines recommend 9–12 months of treatment for children with MDR-TB, regimens may be shorter (6–9 months) in non-severe or longer (9–12 months) in severe cases [1, 3, 16]. Drug adverse events (DAE) are common, possibly due to the limited evidence on dosing and treatment duration in children [3, 4]. According to own experience and South African literature [10], delayed contact investigation and lack of knowledge of MDR-TB contributes to prolonged time-to-treatment.

We hypothesized that the time-to-treatment in children with MDR-pTB was significantly longer than for DS-pTB in Germany due to often missing microbiological results, complex diagnostics, and frequently unknown index. We aimed to determine causes for this putative delay, focusing on possible risk factors for MDR and analyzing epidemiological and clinical characteristics of such cases. Primary endpoint was the time-to-treatment. Secondary endpoints were severity of disease, DAE, and therapy outcome.

## Materials and methods

### Study design

A nationwide retrospective multicenter case–control study, with non-matched control sample from the same centers.

### Ethics approval

The Ethics Committee of Ruhr-University Bochum approved this study (No. 19–6545-BR, approved: 29 May 2019) and waived the need to obtain informed consent from the included patients due to the retrospective nature of this study. The study was registered with the *Deutsches Register Klinischer Studien* (ID: DRKS00023817; registered: 8 September 2020).

### Data collection

Cases were identified through the German Society for Pediatric Pneumology at hospitals in Bochum, Berlin, Hannover, Regensburg, Kaiserslautern, Dinslaken, Soest, Essen, Karlsruhe, Wangen, Erlangen, and Bremen. Data were retrieved from the patients’ medical files and by interviewing the attending physicians. Information was recorded using the Research Electronic Data Capture (REDCap) software (powered by Vanderbilt University, Nashville, Tennessee). This database was established by the Pediatric Tuberculosis Network European Trials Group (ptbnet) [17]. We used the database of the Robert-Koch-Institute (RKI), where TB is notified in Germany, to verify our case numbers.

### Study population

MDR was defined as resistance to isoniazid and rifampin in the child’s or the index`DST [1]. Inclusion criteria were a bacteriologically (culture, PCR) or clinically (symptoms, medical imaging, immunological testing) [17] confirmed tuberculosis in children below 18 years, living in Germany and diagnosed between 2010 and 2020 with informed consent. Children with drug-susceptible TB (DS-pTB) were included in equal proportions from the same centers as cases and acted as controls. Positive immunological testing without clinical, microbiological, or radiological pathology suggestive of tuberculosis was classified as tuberculosis infection (TBI) [1–5]. Children with TBI were assumed as DS or MDR according to their index-patient.

### Epidemiological and clinical characteristics

TB was classified as pulmonary (lung parenchyma, intrathoracic lymph nodes, larynx, trachea, bronchi, pleura), extrapulmonary, or disseminated [5]. Uncontrolled, disseminated disease or complications were criteria for severe disease [18].

Time-to-treatment was defined as the time between first contact with healthcare professionals for the current episode and the date of diagnosis and effective treatment initiation [10]. Reasons for healthcare contact were grouped as contact tracing, migrant screening, or presence of symptoms. Symptoms were prioritized when there were multiple reasons. Country of birth was classified based on WHO disease burden definitions [15]. Refugees, immigrant children and international adoptions counted as migration background. The index case was the person assumed to have infected the child [4].

Clinical history, including previous anti-TB medication and Bacillus Calmette-Guérin (BCG) vaccination, comorbidities, inherited or acquired immunodeficiency, human immunodeficiency virus (HIV) status, and TB signs and symptoms, were collected, and analyzed. Nutritional status was assessed based by the body mass index (BMI) Z-score using Ped(Z) calculator [19] based on the Central European standard values, with Z-scores under –1.96 indicating malnutrition.

### Follow-up

Treatment adherence and completion were monitored, and DAEs were documented throughout treatment and up to two-year follow-up. WHO-defined treatment outcomes for TB included *cured* (treatment completed, clinical symptoms resolved, radiological improvement, sputum culture conversion), *probably cured* (missing culture conversion), *treatment failed* (culture remains positive or missing clinical or radiological improvement), *died*, and *lost to follow-up*. Children with TBI *remained asymptomatic*, *developed TB disease*, or were *lost to follow-up*. Recurrence was possible after treatment completion [4].

### Statistical analysis

Continuous variables are expressed as mean ± standard deviation and were compared using the two-sample *t*-test. Discrete variables are presented as percentages and were compared using the χ^2^ or Fisher’s exact test. Bivariate analysis assessed associations between time-to-treatment and drug-susceptibility. Risk factors for MDR were determined by univariate analyses (odds ratio), while the best factor combination was assessed with the fast-and-frugal decision trees (FFTrees) [20]. Logistic regression was not applied because high interactions between factors were assumed. We chose a dfan algorithm, a high-sensitivity aim, and a goal of 0.6-sensitivity-weighted accuracy. Statistical analysis was performed using R (R Core Team) [21] and Microsoft Excel (Version 16.58, 2022). Two-sided *p* < 0.05 was considered statistically significant.

## Results

### Epidemiology

This study included 52 patients with MDR mycobacteria (24 MDR-pTB, mean age 7.11 years; 28 MDR-pTBI, 7.46 years) and 56 with DS mycobacteria (31 DS-pTB, 7.87 years; 25 DS-pTBI, 6.81 years).

Distribution of sex, household size, and migration background was similar between groups. More children with MDR-pTBI (52%) and MDR-pTB (22%) were born in in the Commonwealth of Independent States (CIS) than children with DS-pTB (13%) or DS-pTBI (0%; *p* < 0.001). Unlike children with DS-pTB (5%), most index cases of children with MDR had drug-resistant mycobacteria (94% MDR-pTB, 100% MDR-pTBI). The annual number of cases with MDR-pTB in this study increased from one in 2010 to five in 2020. In comparison to RKI, we identified more cases because we included children clinically diagnosed as MDR-TB.

More children with DS than MDR mycobacteria received BCG-vaccination (4% MDR-pTB vs. 16% DS-pTB (p < 0.050) and 11% MDR-pTBI vs. 36% DS-pTBI). More children with MDR-pTB than DS-pTB had comorbidities (33% vs. 16%), one child with MDR-pTB was co-infected with HIV (Table 1).

**Table 1:** Clinical data summary of all children included in the study. The participants were grouped into those with tuberculosis disease (TB disease) or tuberculosis infection (TBI) and sub-grouped into multidrug-resistant (MDR) and drug-susceptible (DS) tuberculosis

		**TB disease**			**TBI**		
**Subjects**		**MDR**	**DS**	***p***	**MDR**	**DS**	***p***
	*n*	24	31		28	25	
Male	*n (%)*	13 (54.2%)	15 (48.4%)	0.878	16 (57.1%)	12 (48%)	1.000
Age (years)	*mean (*± *SD)*	7.11 (± 5.4)	7.87 (± 5.6)	0.615	7.46 (± 4.6)	6.81 (± 3.99)	0.586
Age < 5 years	*n (%)*	12 (50%)	13 (41.9%)	0.747	10 (35.7%)	12 (48%)	0.531
**Medical history**							
BMI Z-Score < -1.96	*n/N (%)*	3/19 (15.8%)	7/29 (24.1%)	**0.000**	0/27 (0%)	1/15 (6.7%)	**0.000**
Any previous TB treatment	*n (%)*	4 (16.7%)	4 (12.9%)	0.286	4 (14.3%)	3 (12%)	0.359
BCG vaccination	*n (%)*	1 (4.2%)	5 (16.1%)	**0.036**	3 (10.7%)	9 (36%)	0.062
Comorbidities	*n (%)*	8 (33.3%)	5 (16.1%)	0.201	2 (7.1%)	7 (28%)	**0.011**
Immunodeficiency^a^	*n (%)*	8 (33.3%)	3 (9.7%)	0.097	0 (0%)	1 (4%)	0.218
**Reason for consultation**				0.571			**0.004**
Contact tracing	*n (%)*	9 (37.5%)	8 (25.8%)	0.525	27 (96.4%)	16 (64%)	**0.004**
Screening	*n (%)*	3 (12.5%)	3 (9.7%)	1.000	1 (3.6%)	9 (12%)	**0.004**
Symptoms	*n (%)*	12 (50%)	20 (64.5%)	0.420	0 (0%)	0 (0%)	
**Epidemiology**							
Migration background^b^	*n (%)*	14 (58.3%)	14 (45.2%)	0.683	21 (75%)	15 (60%)	0.500
**Country of birth**^c^	*n*	18	30	**0.008**	27	23	**0.000**
Germany	*n (%)*	4 (22.2%)	13 (43.3%)	0.139	6 (22.2%)	8 (34.8%)	0.324
CIS^d^	*n (%)*	4 (22.2%)	4 (13.3%)	0.424	14 (51.9%)	0 (0%)	**0.001**
Europe^e^	*n (%)*	3 (16.7%)	8 (26.7%)	0.425	6 (22.2%)	8 (34.8%)	0.324
Africa^f^	*n (%)*	6 (33.3%)	3 (10%)	**0.045**	0 (0%)	2 (8.7%)	0.940
Australasia^g^	*n (%)*	1 (11.1%)	2 (6.7%)	0.720	1 (3.7%)	5 (21.7%)	**0.050**
MDR-TB high-burden country^h^	*n (%)*	1 (5.6%)	4 (13.3%)	0.288	9 (33.3%)	0 (0%)	**0.002**
**Index patient**	*n (%)*	16 (66.7%)	21 (67.7%)		28 (100%)	15 (60%)	
Shares home	*n/N (%)*	16/16 (100%)	19/21 (90.5%)	1.000	24/28 (85.7%)	13/14 (92.9%)	0.790
Had DR TB	*n/N (%)*	15/16 (93.8%)	1/21 (4.8%)	**0.000**	28/28 (100%)	0/15 (0%)	**0.000**
DST available	*n/N (%)*	15/16 (93.8%)	14/21 (66.7%)	**0.013**	27/28 (96.4%)	8/15 (53.3%)	**0.004**

*BMI* body mass index, *BCG* Bacillus Calmette-Guérin, *DST* drug susceptibility testing, *SD* standard deviation, *DR* drug resistant, *CIS* Commonwealth of Independent States

*p* < 0.05 is considered as statistically significant and presented in bold

^a^Including human immunodeficiency virus (HIV)

^b^Refugee, immigrant child, international adoption

^c^Only countries where study participants were born

^d^Commonwealth of Independent States (CIS): Azerbaijan, Moldova, Russia

^e^Europe: Italy, the Netherlands, Czech Republic, Hungary, Poland, Albania, Bosnia, Romania, Serbia, Bulgaria, Ukraine, Estonia, Latvia

^f^Africa: Angola, Gabon, Guinea, Somalia, Tunisia, Libya

^g^Australasia: Iraq, Syria, Afghanistan, Australia (Norfolk Island)

^h^MDR-TB high-burden countries: India, China Russian Federation

### Diagnostic management

At the time of diagnosis, the country of birth was known for 75% of those with MDR-pTB, 97% DS-pTB, 96% MDR-pTBI, and 92% DS-pTBI. An index case was identified for two-thirds of the pTB patients, all those with MDR-pTBI, and 60% of those with DS-pTBI, predominantly among household members. The index` DST was available for 94% of those with MDR-pTB, 67% DS-pTB, 96% MDR-pTBI, and 53% DS-pTBI. In TBI-cases without index´ DST drug-susceptibility was presumed based on response to first line treatment.

MDR-pTB and DS-pTB differed in the rates of children with disseminated (21% vs. 0%), and extrapulmonary (25% vs. 13%) disease (*p* = 0.006). Severity of disease correlated with time-to-treatment (r = 0.28, *p* = 0.040) and treatment duration (r = 0.474, *p* = 0.001). Time-to-treatment was strongly correlated with treatment duration (r = 0.839, *p* = 0.001). Most patients with MDR-pTB (71%) were confirmed bacteriologically, over 50% by culture (vs. 61% and 37%, respectively, of those with DS-pTB) (Table 2). More samples were needed to confirm MDR-pTB than DS-pTB (mean 2.8 vs. 1.7 samples; *p* = 0.004). Common sample sources were gastric aspirate (16 MDR; 15 DS), sputum (15 MDR; 13 DS), and bronchoalveolar lavage (13 MDR; 8 DS). Four DS-pTB cases were confirmed by PCR, cultures were either missing or not interpretable due to contamination.

**Table 2:** Diagnostic, treatment regimen, and outcome parameters of all study participants. The patients were grouped into those with tuberculosis disease (TB disease) or TB infection (TBI). and sub-grouped into multidrug-resistant (MDR) and drug-susceptible (DS) TB

		**TB disease**			**TBI**		
**Subject**		**MDR**	**DS**	***p***	**MDR**	**DS**	***p***
	*n*	24	31		28	25	
**Symptoms, clinical presentation**							
Any symptom	*n (%)*	12 (50%)	20(64.5%)	0.420			
**Site of TB**	*n*	24	31	**0.006**			
Only respiratory	*n (%)*	13 (54.2%)	27(87.1%)				
Only extrapulmonary	*n (%)*	6 (25%)	4 (12.9%)				
Multiple	*n (%)*	5 (20.8%)	0 (0%)				
**Diagnosis**							
Symptom duration before diagnosis (days)	*(n) mean (*± *SD)*	(11) 39.14(± 44.5)	(21) 42.45(± 78.2)				
Time-to-treatment (days)^a^	*(n) mean (*± *SD)*	(23) 46.96 (± 64.16)	(31) 10.68 (± 16.24)	**0.004**	(27) 40.11 (± 34.9)	(25) 3.88 (± 4.23)	**0.000**
Positive immunological testing till treatment start	*(n) mean (*± *SD)*	(22) 45.1 (± 95.2)	(28) 12.3 (± 53.9)		(24) 29.5 (± 31.3)	(25) 4.4 (± 3.7)	
**Confirmation**				0.872			**0.000**
Bacteriological	*n (%)*	17 (70.8%)	19 (61.3%)		0 (0%)	0 (0%)	
Clinical	*n (%)*	7 (29.2%)	11 (35.5%)		28 (100%)	25 (100%)	
**Bacteriological confirmation**	*n*	17	19	0.272			
By culture	*n (%)*	9 (52.9%)	7 (36.8%)				
By PCR	*n (%)*	0 (0%)	4 (21.1%)				
By culture + PCR	*n (%)*	8 (47.1%)	8 (42.1%)				
**Outcome**							
Drug adverse events	*n/N (%)*	11/23 (47.8%)	4/31 (12.9%)	0.180	5/28 (17.9%)	2/25 (8%)	0.386
Sequelae	*n/N (%)*	5/23 (21.7%)	4/30 (13.3%)	0.478			
Reinfection	*n/N (%)*	0/19 (0%)	1/30 (3.3%)	1.000			
Developed disease	*n (%)*				1 (3.6%)	0 (0%)	0.340
Lost to follow-up	*n (%)*	0 (0%)	1 (3.23%)		0 (0%)	3 (12%)	0.059
Positive outcome^b^	*n/N (%)*	14/19 (73.7%)	24/30 (80%)	1.000	27/28 (96.4%)	22/25 (88%)	0.333

*p* < 0,05 is considered as statistically significant and presented in bold

^a^time-to-treatment = time from the first healthcare contact for the current episode to the day on which the diagnosis was made and therapeutic regimen was decided

^b^Cured, probably cured, or remained asymptomatic once treatment completed

### Risk factors for MDR mycobacterial infection

**Fig. 1:**
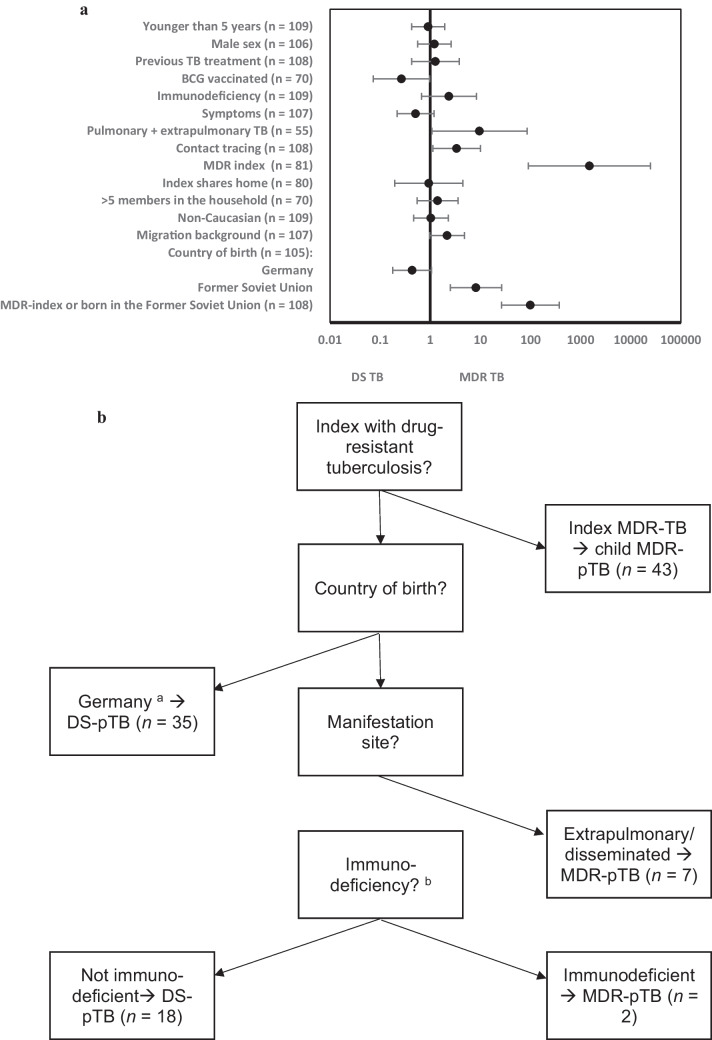
**a** Risk factors for multidrug resistance in children with tuberculosis (ordinate). Data are presented in a logarithmic scale, with odds ratios and confidence intervals; BCG, Bacillus Calmette-Guérin. **b** Fast-and-frugal decision tree based on risk factors with the highest odds ratio to identify pediatric multidrug-resistant tuberculosis cases [*n* = 108; 52 with multidrug-resistant tuberculosis (MDR-TB) and 56 with drug-susceptible tuberculosis (DS-TB)]. Three false-negative patients with MDR-TB were detected. Sensitivity was 100%, and specificity was 95%

Risk factors for MDR were an index case with MDR mycobacteria, birth in an MDR mycobacterial high-burden country, and disseminated disease. The optimal decision tree had 100% sensitivity and 95% specificity (53 of 56 with MDR) based on the index` DST, country of birth, site of TB, and immune competence. We could classify 72% of our study population into the MDR or control group according to their index` drug susceptibility and country of birth. In the remaining, site of TB and immune competence fitted best as decision factors (Fig. 1).

### Time-to-treatment

**Fig. 2:**
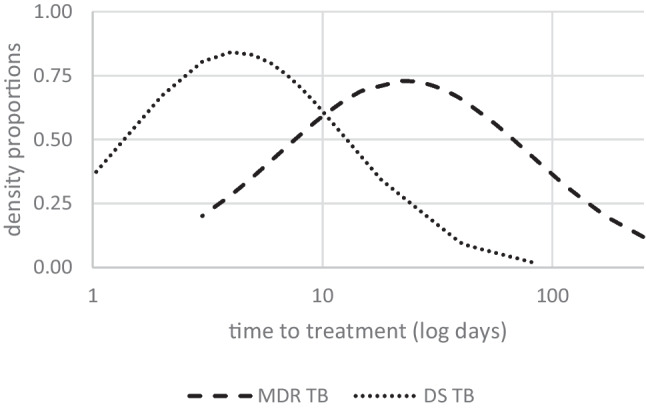
Density proportions (ordinate) of the time-to-treatment (in days, logarithmic scale, abscissa) of multidrug-resistant tuberculosis (MDR TB) and drug-susceptible tuberculosis (DS TB). Density probabilities were estimated using normal distribution

It took over four times longer to treat MDR-pTB than DS-pTB (mean, median [range] in days: 46.96, 26 [3–248] vs. 10.68, 5.5 [0–81]), irrespective of the patient’s age (Fig. 2).

MDR-pTBI time-to-treatment was over ten times longer than DS-pTBI (40.11, 25 [0–188] vs. 3.88, 3 [0–18]). All patients with TBI were diagnosed based on their index` DST (*n* = 27; mean time-to-diagnosis, 40.1 days). Two were immediately diagnosed as MDR, the others index cases were presumed as DS until DST-results were available and partly treated with isoniazid.

**Fig. 3:**
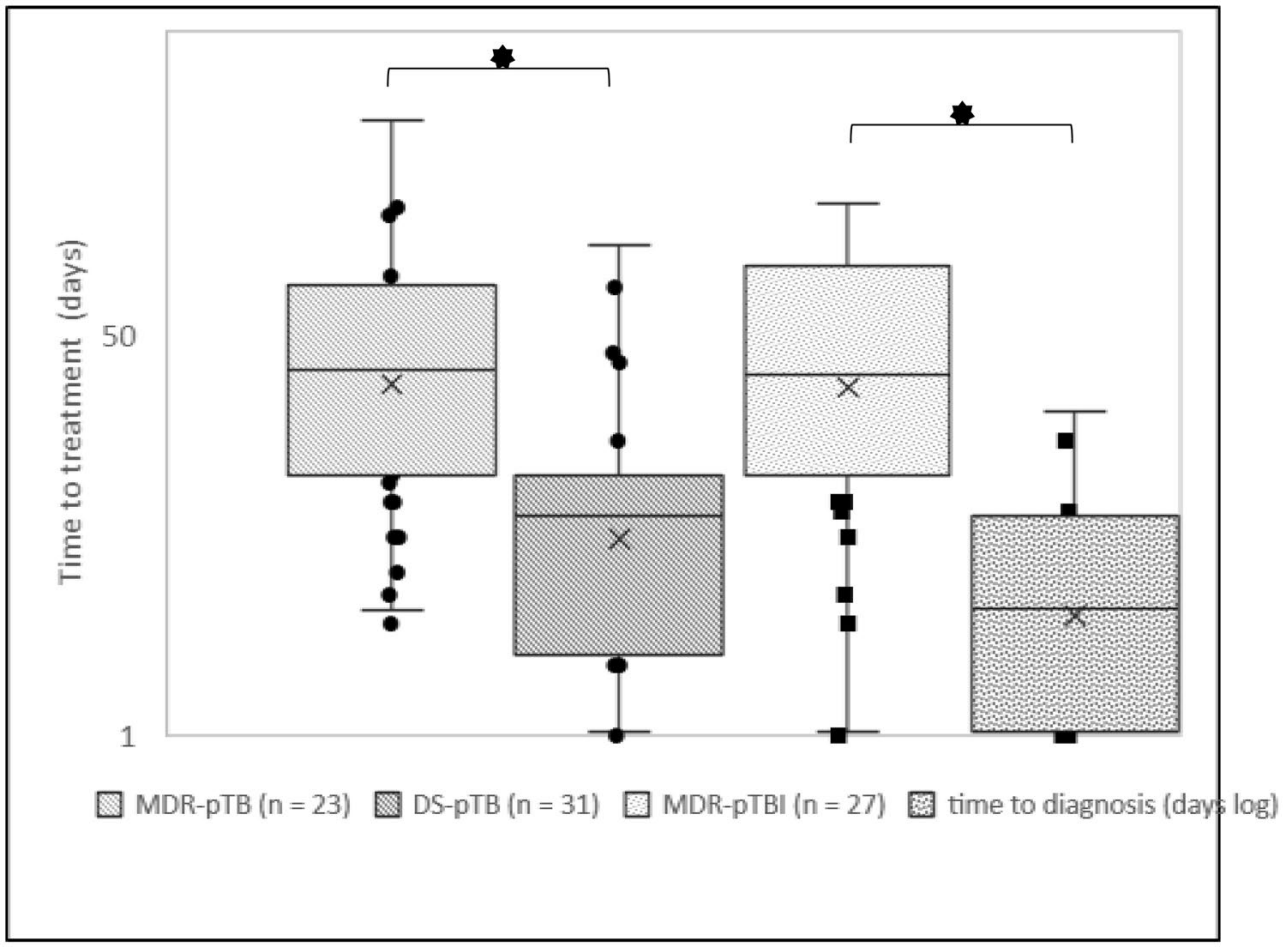
The time between the first clinical contact and treatment (days, logarithmic scale). Presented are the means (×), medians (horizontal line), quartiles, and individual values of children with multidrug-resistant (MDR) and drug-susceptible (DS) tuberculosis (TB) disease and tuberculosis infection (TBI). * *p* < 0.05

**Fig. 4:**
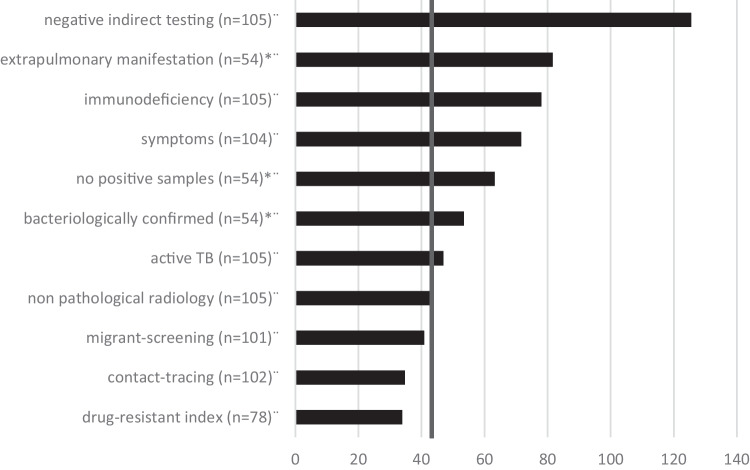
Factors (ordinate) affecting the time to tuberculosis (TB) treatment (abscissa, days) in children with multidrug-resistant (MDR) TB (bars). Mean time to MDR-TB treatment (vertical line) was 43.26 days. Information is based on the available *n* as indicated. * Only available for TB disease

Time-to-treatment of cases with MDR-pTB and DS-pTB varied over the observational period (Table 2; Figs. 3 and 4). Diagnosis took longest in children with symptomatic TB and unidentified index case. Contact tracing, migrant screening, and an index with MDR mycobacteria contribute to shorter time-to-treatment (Fig. 4). Immunologic evidence of TB became available early, but targeted therapy initiation took long (mean ± standard deviation (SD) in days: 45 ± 95 MDR-pTB vs. 12 ± 54 DS-pTB) (Table 2).

### Therapy regimen

Children with MDR-pTB were treated significantly longer (17.9 months ± 4.7 vs. 7.8 ± 4.7) and with more drugs (4.7 ± 1.9 vs. 3.7 ± 0.5) than DS-pTB. 54% of those with MDR-pTB received injectables. Treatment duration decreased from 2010 (mean, 27 months) to 2020 (mean, 11 months). Pharmacotherapy for patients with MDR-pTB frequently included linezolid (71%), moxifloxacin (67%), and cycloserine (67%), new drugs were bedaquiline (*n* = 7, no DAE) and delamanid (*n* = 4, 1 DAE).

**Fig. 5:**
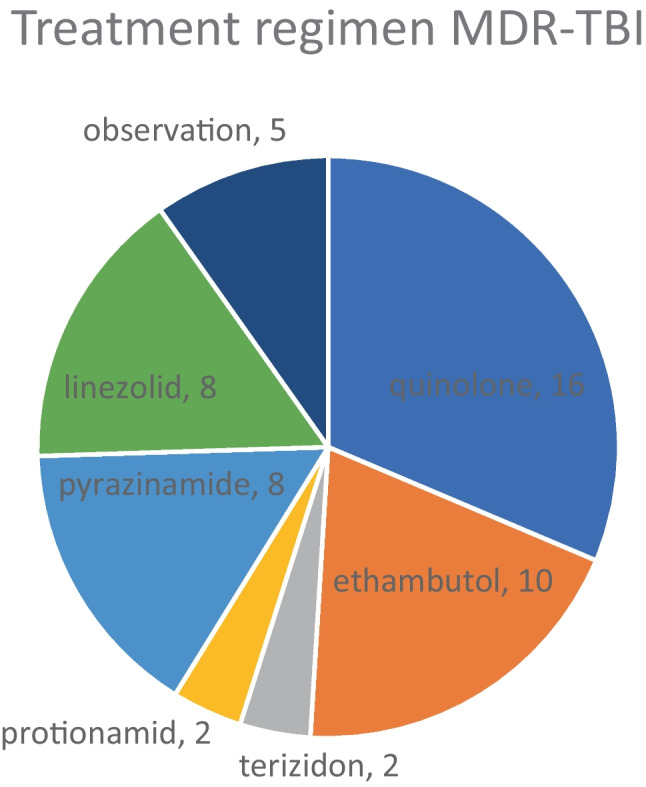
Treatment regimen for 28 children with multidrug-resistant tuberculosis infection (MDR-TBI)

All cases with DS-pTBI received isoniazid and rifampin for three months. Among children with MDR-pTBI, five were monitored without medication, while the others received DST-adapted regimens with an average duration of nine months (Fig. 5).

### Adverse events and outcome

**Fig. 6:**
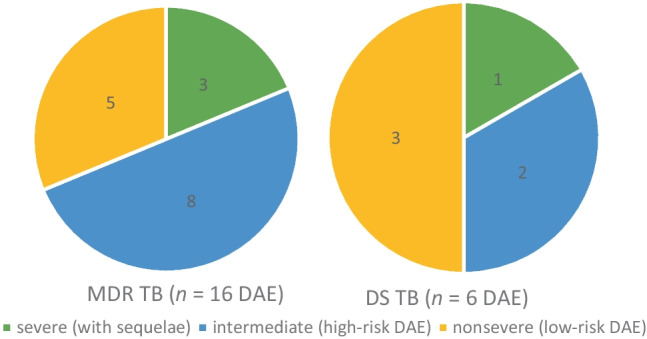
Severity of drug adverse events (DAE) in children with mutlidrug-resistant tuberculosis (MDR-TB; left) and drug-sensitive tuberculosis (DS-TB; right). Hearing and vision impairment, hypothyroidism, neutropenia, thrombozytopenia, and hepatitis were defined as high-risk DAE, while symptoms like nausea as low-risk DAE

DAEs, including hearing or vision impairment, hypothyroidism, hepatitis, neutropenia, and nausea, occurred in many cases with MDR-pTB. Neutropenia and gastrointestinal DAEs were twice as frequent in MDR-pTBI as in DS-pTBI. DAEs were primarily associated with linezolid (*n* = 5), protionamide (*n* = 4), and amikacin (*n* = 4). Drugs frequently used without DAE were clofazimine, cycloserine and moxifloxacin, but not ethambutol (*n* = 4, 2 DAE) (Fig. 6).

Vision impairment occurred in two MDR and one DS-TB case who were treated with ethambutol, linezolid, and PAS. Hearing impairment occurred in four patients, each of them received injectable aminoglycosides (amikacin). Most children recovered (cured or probably cured) from TB after complete treatment (MDR-pTB 74%, DS-pTB 80%, MDR-pTBI 96%, DS-pTBI 88%), no child died. Nine children developed sequelae (MDR-pTB 22%, DS-pTB 13%), which were permanent loss of hearing (*n* = 2), respiratory (*n* = 4) and osteoarticular impairment (*n* = 3). Longer treatment made sequelae more likely (r = 0.358, *p* = 0.012). Reinfection occurred in one child with DS-pTB.

One child with MDR-pTBI developed TB disease, and three with DS-pTBI were lost to follow-up. The other children with TBI remained asymptomatic (Table 2).

## Discussion

Despite recent improvements in the diagnostic and therapeutic management of MDR-pTB, our study in a low-incidence country confirms diagnostic latency, which is associated with a higher risk of advanced disease, long treatment duration and adverse effects. The main risk factors for MDR-TB were an index with MDR-TB and birth in MDR-TB high-incidence countries like the CIS.

### Time-to-treatment

In MDR-pTB cases, time to effective treatment initiation was four times longer than in DS-pTB. Immunologic evidence of TB usually became available early, but initiation of targeted therapy was often postponed, showing a wide interindividual range. Diagnosis took the longest in children with symptomatic TB disease and an unidentified index case. Low TB incidence among children in Germany and the even lower incidence of MDR-TB might contribute to the delayed diagnosis. When an adult is diagnosed with TB, detailed contact investigation is essential to detect exposed children early. Children often have paucibacillary disease and microbiological confirmation and DST is difficult. Three cases with MDR (1 pTB, 2 pTBI) and a known MDR index were diagnosed as MDR immediately. When the index case was unknown, diagnosis of drug resistance often took long and required repeated microbiological sampling due to the paucibacillary disease and infrequent use of genotypic confirmation. Children with MDR-pTBI were verified by the index DST, which was not always immediately available and only a surrogate of the child’s DST.

In 2014, a South African study found that the time from sputum collection to diagnosis using GeneXpert in adults was significantly shorter than by culture [22]. In our pediatric study population, GeneXpert or other nucleic-acid amplification techniques were not applied regularly. Diagnosis was established by culture in 53% of the cases with MDR-pTB (37% DS-pTB). The remaining cases were diagnosed clinically and through index investigation. In an American study population, 25% were bacteriologically confirmed [23], while 70% of the participants in a British study had positive culture or polymerase chain reaction (PCR) [24]. More extensive use of molecular tests (GeneXpert, Line Probe assays, NGS) might have facilitated the diagnosis of MDR-pTB resulting in earlier initiation of treatment [25, 26].

Schaaf et al. observed an extended time-to-treatment among patients with MDR-pTB in South Africa in 2003 (median, 283 days), but shorter in children with a known source case (median, 246 days) or a known MDR-TB index case (median, 2 days) [10]. Time-to-treatment varied from two days to 46 months in a meta-analysis from 2012 [27]. To shorten this interval, timely contact investigations and better cooperation between public health authorities and pediatricians are needed to identify index patients or transmission dynamics [28].

### Risk factors for MDR

Risk factors for MDR were exposure to an index with MDR-TB or birth in a CIS country. A flowchart for cases with pTB at high risk for MDR revealed that primary contact tracing contributed greatly to identifying those with MDR.

An index case was identified in 67% of our patients with MDR-pTB, 94% of whom had MDR-TB, and all were household members. All contacts in the American study [23] and 21/39 in the South African study had MDR-TB [10]. High prevalence of TB infection among MDR-TB household members was observed in high-burden countries [29]. Estimated 25,000–32,000 children develop MDR-TB disease each year but only 3–4% of them receive treatment. Effective contact investigations around adult MDR-TB might contribute to identify twelve times more MDR-pTB cases [30]. These cases with MDR-pTB could be treated according to their index` DST since equivalent resistance profiles among household members were assumed [31, 32], shortening the time to adequate treatment [10]. Due to the assumption that in a low-incidence setting every child with TBI and an index with MDR had MDR-pTBI, 100% of the index cases of MDR-pTBI were identified.

Other studies identified birth in a foreign country and previous TB treatment as risk factors for MDR-TB in low-incidence countries [33]. In Germany, many children with TB had a migration background, involving language and cultural barriers. Asylum seekers in Germany have limited access to healthcare services [28]. The groups differed significantly with respect to countries of birth: most patients with MDR-pTB were born in the CIS, while most patients with DS-pTB were German-born. Glasauer et al. described high rates of drug resistance among German-born and foreign-born TB-patients, particularly high in patients born in Russia (33%) [33].

One third of children in our study were BCG-vaccinated, significantly more children with DS mycobacteria than MDR, depending on country-specific WHO recommendations. BCG-vaccination is neither effective in preventing DS- or MDR-TB. In high-burden countries children should receive a single BCG-dose, significantly reducing their risk of severe TB. In Germany BCG-vaccination was abandoned in 1998 due to low-incidence. So, children born in high-burden countries who immigrate to Germany are not vaccinated [1, 34].

### Treatment

Patients with MDR-pTB were mainly treated with linezolid (71%), moxifloxacin (67%), and cycloserine (67%), according to WHO [1]. Based on previous recommendations, 54% received injectables which cause a high rate of adverse effects. In our study, hearing impairment occurred four times in patients receiving amikacin, two children did not recover.

New drugs (bedaquiline and delamanid) were approved for TB treatment in Europe during this study [35]. Linezolid effectiveness in patients with drug-resistant TB was documented in several studies [36]. The efficacy of a reduced dose has been evaluated since linezolid is associated with frequent DAEs [37].

There is low evidence for preventive treatment or observation of MDR-TB contacts [38]. Based on observational studies [39], WHO suggests levofloxacin in combination with ethambutol or ethionamide for 6 months. The combination of levofloxacin and pyrazinamide is not recommended due to high risk of adverse events [1]. Clinical trials for levofloxacin in other combination [40] or monotherapy ((V-QUIN MDR-TB, Vietnam [41]), TB-CHAMP (children < 5 years, South Africa) and PHOENIx (delamanid, international)) are ongoing [42]. In a South African study a 6-months-regimen with ofloxacin, ethambutol and isoniazid was well tolerated and only 4% of pediatric MDR contacts developed TB [43].

### Adverse events and outcome

Five of 17 children treated with linezolid in our study had DAEs, none of seven treated with bedaquiline, and one of four treated with delamanid. Recommendations and treatment have changed over the observation period. We found no difference in treatment outcomes between MDR-pTB and DS-pTB, but patients with MDR-pTB required a longer treatment, and transient or permanent DAEs occurred in almost half of them. The DAEs in our study population (hearing or vision impairments, hypothyroidism, hepatitis, neutropenia, nausea) are commonly described in MDR-pTB [27]. Two children in our study experienced permanent hearing loss, probably caused by aminoglycoside treatment [28]. Treatment was successful in 74% of MDR-TB cases in our study, comparable to 78% in a meta-analysis [44].

No deaths were reported in our study, whereas 4/39 children with MDR-pTB died in the South African study [10]. Two children with poor outcomes in the South African study and one in our study were infected with HIV. The pTB mortality rate in India is 5.4%, mainly due to drug resistance, delayed presentation to healthcare, or disseminated disease [45]. Outcomes in developing countries are worse than in developed countries [46].

### Further findings

Most cases with pTB in Europe were not diagnosed in specialized hospitals [15, 28]. Patients with MDR-TB in the US are cared for in public health settings [47]. Although hospitals treating children with MDR-pTB in Germany differed in their specialization level, no differences in outcomes were detected [28]. All but three centers treated adult index patients and children separately. We found more cases than reported by the RKI because they only considered culture-confirmed TB disease cases in children under the age of 15 as pTB [6].

### Limitations

Although we aimed to include all children diagnosed with MDR-pTB in Germany from 2010 to 2020, the sample size was small corresponding to a low-incidence setting.

Selection bias could not be excluded, particularly since diagnosis of TBI relied on an index case and was assumed as MDR-TBI according to the index´ DST. The retrospective study design was another limitation. Although all original findings and documentation were studied, some data remained incomplete. Moreover, we could not include cases with DS-pTB from all participating centers, but similar numbers of controls were included from hospitals with many MDR cases over the same period.

### Conclusion

Children with close contact to an MDR index case or from MDR-TB-high-incidence countries are at higher risk for developing MDR-TB. Early identification of potential MDR index cases, repeated collection of microbiological samples, and DST in children from high-burden MDR-TB countries are essential for timely diagnosis and treatment, reducing the severity of disease and treatment side effects. Bacteriological confirmation, including PCR-based DST, should always be attempted in children whose index had MDR-TB or who were born in a high-incidence country. Initial treatment should follow the index` DST and resistance profile of the country of birth. The effect of contact tracing requires evaluation in further prospective studies.

## Data Availability

Data is accessible via the ptbnet database and is available on request.

## Abbreviations

AWMF: German Association of the Scientific Medical Societies
BCG: Bacillus Calmette-Guérin
BMI: Body Mass Index
CIS: Commonwealth of Independent States
DAE: Drug adverse events
DS-pTB: Pediatric drug-susceptible TB
DS-TB: Drug-susceptible TB
DST: Drug susceptibility testing
FFTrees: Fast-and-frugal decision trees
HIV: Human immunodeficiency virus
TBI: Tuberculosis infection
MDR-pTB: Pediatric multidrug-resistant tuberculosis
MDR-TB: Multidrug-resistant tuberculosis
PCR: Polymerase chain reaction
ptbnet: Pediatric Tuberculosis Network European Trials Group
REDCap: Research Electronic Data Capture
RKI: Robert Koch Institute
SD: Standard deviation
TB: Tuberculosis
WHO: World Health Organization

## References

[1] World Health Organization (2022) WHO consolidated guidelines on tuberculosis: Module 5: management of tuberculosis in children and adolescents. World Health Organization 35404556

[2] Feiterna-Sperling C, Brinkmann F, Adamczick C, Ahrens F, Barker M, Berger C, Berthold LD, Bogyi M, von Both U, Frischer T, Haas W, Hartmann P, Hillemann D, Hirsch FW, Kranzer K, Kunitz F, Maritz E, Pizzulli A, Ritz N, Schlags R, Spindler T, Thee S, Weizsäcker K (2017). Consensus-based guidelines for diagnosis, prevention and treatment of tuberculosis in children and adolescents - a guideline on behalf of the German Society for Pediatric Infectious Diseases (DGPI). Pneumologie.

[3] Furin J, Seddon J, Becerra M (2021) Management of drug-resistant tuberculosis in children: a field guide. Boston, USA. The Sentinel Project for Pediatric Drug-Resistant Tuberculosis Fifth edition

[4] Seddon J, Perez-Velez C, Schaaf H, Furin JJ, Marais BJ, Tebruegge M, Detjen A, Hesseling AC, Shah S, Adams LV, Starke JR, Swaminathan S, Becerra MC, Sentinel Project on Pediatric Drug-Resistant Tuberculosis (2013). Consensus statement on research definitions for drug-resistant tuberculosis in children. J Pediatric Infect Dis Soc.

[5] Seddon J, Johnson S, Palmer M, van der Zalm MM, Lopez-Varela E, Hughes J, Schaaf HS (2021). Multidrug-resistant tuberculosis in children and adolescents: current strategies for prevention and treatment. Expert Rev Respir Med.

[6] Brodhun B, Altmann D, Hauer B (2021) Bericht zur Epidemiologie der Tuberkulose in Deutschland für 2020. Robert Koch Institut, Berlin, Germany

[7] Brodhun B, Altmann D, Hauer B (2020) Bericht zur Epidemiologie der Tuberkulose in Deutschland für 2019. Robert Koch Institut, Berlin, Germany

[8] Lange C, Abubakar I, Alffenaar J-W, Bothamley G, Caminero JA, Carvalho AC, Chang KC (2014). Management of patients with multidrug-resistant/extensively drug-resistant tuberculosis in Europe: a TBNET consensus statement. Eur Respir J.

[9] Dunn JJ, Starke JR, Revell PA (2016). Laboratory diagnosis of Mycobacterium tuberculosis infection and disease in children. J Clin Microbiol.

[10] Schaaf H, Shean K, Donald P (2003). Culture confirmed multidrug resistant tuberculosis: diagnostic delay, clinical features, and outcome. Arch Dis Child.

[11] Graham SM (2011). Treatment of paediatric TB: revised WHO guidelines. Paediatr Respir Rev.

[12] Bossù G, Autore G, Bernardi L, Buonsenso D, Migliori GB, Esposito S (2022) Treatment options for children with multi-drug resistant tuberculosis. Expert Rev Clin Pharmacol:1–11 10.1080/17512433.2023.2148653 36378271

[13] CLSI (2018) Susceptibility testing of mycobacteria, Nocardia spp., and other aerobic actinomycetes. CLSI standard M24. Clinical and Laboratory Standards Institute, Wayne, PA 31339680

[14] Khan MA, Bilal W, Asim H, Rahmat ZS, Essar MY, Ahmad S (2022). MDR-TB in Pakistan: Challenges, efforts, and recommendations. Ann Med Surg (Lond).

[15] World Health Organization (2021) Global Tuberculosis Report 2021. World Health Organization, Geneva

[16] Huynh J, Thwaites G, Marais B, Schaaf H (2020). Tuberculosis treatment in children: the changing landscape. Paediatr Respir Rev.

[17] Kampmann B (2009) Paediatric Tuberculosis Network European Trialgroup (ptbnet) [ptbnet web site]

[18] Wiseman CA, Gie RP, Starke JR, Schaaf HS, Donald PR, Cotton MF, Hesseling AC (2012). A proposed comprehensive classification of tuberculosis disease severity in children. Pediatr Infect Dis J.

[19] Gräfe D (2021) Ped(z) Paediatric Calculator. Leipzig, Germany

[20] Phillips ND, Neth H, Woike JK, Gaissmaier W (2017). FFTrees: a toolbox to create, visualize, and evaluate fast-and-frugal decision trees. Judgm Decis Mak.

[21] R Core Team (2021) R: A language and environment for statistical computing. R Foundation for Statistical Computing. Vienna, Austria

[22] Iruedo J, O'Mahony D, Mabunda S, Wright G, Cawe B (2017). The effect of the Xpert MTB/RIF test on the time to MDR-TB treatment initiation in a rural setting: a cohort study in South Africa's Eastern Cape Province. BMC Infect Dis.

[23] Smith SE, Pratt R, Trieu L, Barry PM, Thai DT, Ahuja SD, Shah S (2017). Epidemiology of pediatric multidrug-resistant tuberculosis in the United States, 1993–2014. Clin Infect Dis.

[24] Shah T, Williams B, Langer D, Mitchell H, Togo A, Kreins AY, Caddle L, Das S, Lutkin M, Seddon JA (2015). A retrospective analysis of paediatric tuberculosis diagnosis in London: room for improvement?. Arch Dis Child.

[25] Peralta G, Barry P, Pascopella L (2016). Use of nucleic acid amplification tests in tuberculosis patients in California, 2010–2013. Open Forum Infect Dis.

[26] Roya-Pabon CL, Perez-Velez CM (2016). Tuberculosis exposure, infection and disease in children: a systematic diagnostic approach. Pneumonia (Nathan).

[27] Ettehad D, Schaaf H, Seddon J, Cooke G, Ford N (2012). Treatment outcomes for children with multidrug-resistant tuberculosis: a systematic review and meta-analysis. Lancet Infect Dis.

[28] Joean O, Thiele T, Schütz K, Schwerk N, Sedlacek L, Kalsdorf B, Baumann U, Stoll M (2020). Multidrug-resistant *Mycobacterium tuberculosis*: a report of cosmopolitan microbial migration and an analysis of best management practices. BMC Infect Dis.

[29] Gupta A, Swindells S, Kim S, Hughes M, Naini L, Wu X, Dawson R (2020). Feasibility of identifying household contacts of rifampin-and multidrug-resistant tuberculosis cases at high risk of progression to tuberculosis disease. Clin Infect Dis.

[30] Jenkins H, Yuen C (2018). The burden of multidrug-resistant tuberculosis in children. Int J Tuberc Lung Dis.

[31] Lange C, Dheda K, Chesov D, Mandalakas A, Udwadia Z, Horsburgh C (2019). Management of drug-resistant tuberculosis. Lancet.

[32] Chiang S, Brooks M, Jenkins H, Rubenstein D, Seddon J, van de Water B, Lindeborg M, Becerra M, Yuen C (2021). Concordance of drug-resistance profiles between persons with drug-resistant tuberculosis and their household contacts: a systematic review and meta-analysis. Clin Infect Dis.

[33] Glasauer S, Altmann D, Hauer B, Brodhun B, Haas W, Perumal N (2019). First-line tuberculosis drug resistance patterns and associated risk factors in Germany, 2008–2017. PLoS ONE.

[34] Xu Y, Li Q, Zhu M, Wu X, Wang D, Luo J, Li Y, Zhong J, Zeng P (2020). The epidemiological characteristics and profile of drug-resistant tuberculosis among children with tuberculosis in Sichuan, China, 2015–2018: a retrospective study. Medicine (Baltimore).

[35] Masini T, Hauser J, Kuwana R, Nhat Linh N, Jaramillo E (2018). Will regulatory issues continue to be a major barrier to access to bedaquiline and delamanid?. Eur Respir J.

[36] Lifan Z, Sainan B, Feng S, Siyan Z, Xiaoqing L (2019). Linezolid for the treatment of extensively drug-resistant tuberculosis: a systematic review and meta-analysis. Int J Tuberc Lung Dis.

[37] Bolhuis M, van der Werf T, Kerstjens H, de Lange W, Alffenaar J, Akkerman O (2019). Treatment of multidrug-resistant tuberculosis using therapeutic drug monitoring: first experiences with sub-300 mg linezolid dosages using in-house made capsules. Eur Respir J.

[38] European Centre for Disease Prevention and Control (ECDC) (2012) Management of contacts of MDR TB and XDR TB patients, Stockholm, Sweden

[39] Marks SM, Mase SR, Morris SB (2017). Systematic review, meta-analysis, and cost-effectiveness of treatment of latent tuberculosis to reduce progression to multidrug-resistant tuberculosis. Clin Infect Dis.

[40] Seddon J, Fred D, Amanullah F, Schaaf H, Starke J, Keshavjee S, Burzynski J, Furin J, Swaminathan S, Becerra M (2015) Post-exposure management of multidrug-resistant tuberculosis contacts: evidence-based recommendations. Harvard Medical School Center for Global Health Delivery–Dubai., Dubai, United Arab Emirates

[41] Fox GJ, Nguyen CB, Nguyen TA, Tran PT, Marais BJ, Graham SM, Nguyen BH, Velen K, Dowdy DW, Mason P, Britton WJ, Behr MA, Benedetti A, Menzies D, Nguyen VN, Marks GB (2020). Levofloxacin versus placebo for the treatment of latent tuberculosis among contacts of patients with multidrug-resistant tuberculosis (the VQUIN MDR trial): a protocol for a randomised controlled trial. BMJ Open.

[42] Kherabi Y, Tunesi S, Kay A, Guglielmetti L (2022) Preventive Therapy for Contacts of Drug-Resistant Tuberculosis. Pathogens 11 10.3390/pathogens11101189 PMC9609446 36297246

[43] Seddon JA, Hesseling AC, Finlayson H, Fielding K, Cox H, Hughes J, Godfrey-Faussett P, Schaaf HS (2013). Preventive therapy for child contacts of multidrug-resistant tuberculosis: a prospective cohort study. Clin Infect Dis.

[44] Harausz E, Garcia-Prats A, Law S, Schaaf H, Kredo T, Seddon J, Menzies D (2018). Treatment and outcomes in children with multidrug-resistant tuberculosis: a systematic review and individual patient data meta-analysis. PLoS Med.

[45] Sharma S, Sarin R, Sahu G, Shukla G (2020). Demographic profile, clinical and microbiological predictors of mortality amongst admitted pediatric TB patients in a tertiary referral tuberculosis hospital. Indian J Tuberc.

[46] Tola H, Khadoura K, Jimma W, Nedjat S, Majdzadeh R (2020). Multidrug resistant tuberculosis treatment outcome in children in developing and developed countries: a systematic review and meta-analysis. Int J Infect Dis.

[47] Feja K, McNelley E, Tran C, Burzynski J, Saiman L (2008). Management of pediatric multidrug-resistant tuberculosis and latent tuberculosis infections in New York City from 1995 to 2003. Pediatr Infect Dis J.

